# Identification of genetic mechanisms of non-isolated auditory neuropathy with various phenotypes in Chinese families

**DOI:** 10.1186/s13023-025-03540-7

**Published:** 2025-01-08

**Authors:** Yang Cao, Xiaolong Zhang, Lan Lan, Danyang Li, Jin Li, Linyi Xie, Fen Xiong, Lan Yu, Xiaonan Wu, Hongyang Wang, Qiuju Wang

**Affiliations:** 1 Senior Department of Otolaryngology Head and Neck Surgery, The 6th Medical Center of Chinese PLA General Hospital, Chinese PLA Medical School, Beijing, 100048 China; 2 State Key Laboratory of Hearing and Balance Science, Beijing, 100853 China; 3 National Clinical Research Center for Otolaryngologic Diseases, Beijing, 100853 China

**Keywords:** Non-isolated auditory neuropathy, Hereditary deafness, *FDXR*, *TWNK*, Syndrome

## Abstract

**Background:**

Non-isolated auditory neuropathy (AN), or syndromic AN, is marked by AN along with additional systemic manifestations. The diagnostic process is challenging due to its varied symptoms and overlap with other syndromes. This study focuses on two mitochondrial function-related genes which result in non-isolated AN, *FDXR* and *TWNK*, providing a summary and enrichment analysis of genes associated with non-isolated AN to elucidate the genotype-phenotype correlation and underlying mechanisms.

**Methods:**

Seven independent Chinese Han patients with mutations in *FDXR* and *TWNK* underwent comprehensive clinical evaluations, genetic testing, and bioinformatics analyses. Diagnostic assessments included auditory brainstem response and distortion product otoacoustic emissions, supplemented by other examinations. Whole exome sequencing and Sanger sequencing validated genetic findings. Pathogenicity was assessed following American College of Medical Genetics and Genomics guidelines. Genes associated with non-isolated AN were summarized from prior reports, and functional enrichment analysis was conducted using Gene Ontology databases.

**Results:**

A total of 11 variants linked to non-isolated AN were identified in this study, eight of which were novel. Patients’ age of hearing loss onset ranged from 2 to 25 years, averaging 11 years. Hearing loss varied from mild to profound, with 57.1%(4/7) of patients having risk factors and 71.4%(5/7) exhibiting additional systemic symptoms such as muscle weakness, ataxia, and high arches. Functional enrichment analysis revealed that genes associated with non-isolated AN predominantly involve mitochondrial processes, affecting the central and peripheral nervous, musculoskeletal, and visual systems.

**Conclusion:**

This study identifies novel mutations in *FDXR* and *TWNK* that contribute to non-isolated AN through mitochondrial dysfunction. The findings highlight the role of mitochondrial processes in non-isolated AN, suggesting potential relevance as biomarkers for neurodegenerative diseases. Further research is required to explore these mechanisms and potential therapies.

**Graphical Abstract:**

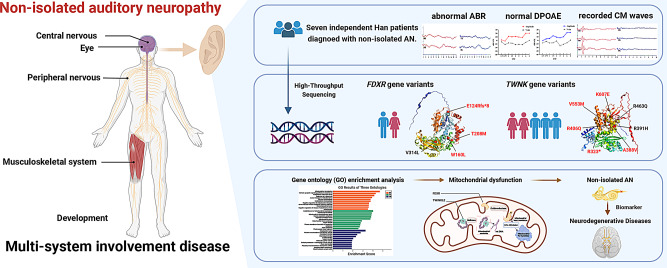

**Supplementary Information:**

The online version contains supplementary material available at 10.1186/s13023-025-03540-7.

## Introduction

Auditory neuropathy (AN) is characterized by the ability to hear sounds without understanding their meaning. The otoacoustic emission (OAE) and/or cochlear microphonic (CM) are preserved but the auditory brainstem response (ABR) is abnormal or absent, which indicates the abnormal auditory nerve function and normal outer hair cell function [1, 2]. With researchers’ deepening understanding of AN, its concept gradually expands to an auditory information processing disorder caused by the malfunctioning of inner hair cells (IHCs), IHC ribbon synapses, spiral ganglion cells or the demyelination and axonal loss of the auditory nerve fibers and their targets in the cochlear nucleus [3, 4]. Regarding whether AN is the sole manifestation, it can be classified into isolated AN and non-isolated AN.

Non-isolated AN, also known as syndromic AN, refers to the clinical phenotype of AN accompanied by other systemic symptoms like central or peripheral neurological pathologies. Manifestations may include muscle weakness and atrophy caused by motor neuron diseases, skeletal anomalies, visual problems, and neurological deficits. Previous studies have shown that more than one-third of patients with AN exhibit peripheral neuropathy [5]. Typical syndromes include Charcot-Marie-Tooth syndrome, Friedreich’s ataxia, Mohr-Tandberg syndrome, Autosomal-dominant optic atrophy and others [5, 6].

The diagnosis of non-isolated AN is complex due to diverse symptoms and overlap with other syndromes. Previous studies have suggested that genetic factors contribute to AN in 31.5% of cases, underscoring the significance of investigating genetic mechanisms in these patients [7]. With advances in genetic testing techniques, more genes are gradually being found to be associated with the onset of AN. In our previous study [7], among the 23 genes identified and classified as pathogenic or likely pathogenic, genes such as *AIFM1*, *TIMM8A*, and *ATP1A3* were found to be associated with the clinical manifestations of auditory neuropathy, accompanied by other systemic symptoms [8–11]. So far, various types of AN, including unilateral and bilateral as well as infantile and non-infantile have been discussed, but further research on isolated versus non-isolated AN is still necessary.

Mitochondrial function significantly influences the cellular energy metabolism and may directly contribute to the pathogenesis of non-isolated AN through its dysfunction. Among the genes associated with non-isolated AN, the *FDXR* and *TWNK* genes have recently garnered attention due to their critical roles in mitochondrial function [12, 13]. *FDXR* gene, encoding the sole ferredoxin reductase implicates in the biosynthesis of iron-sulfur (Fe-S) clusters and heme formation within the human organism, is involved in electron transport in mitochondrial respiration [14, 15]. *TWNK* gene, which encodes the Twinkle protein, exhibits DNA helicase activity and plays a crucial role in the replication and maintenance of mitochondrial DNA (mtDNA). It serves as a key regulator of mtDNA copy number in mammals [16–19].

In this study, we recruited seven independent patients diagnosed with non-isolated AN due to *FDXR* and *TWNK* gene mutations, identified their genotypic and phenotypic characteristics through clinical analysis and genetic testing. Eight of the eleven identified variants were novel, thereby expanding the genomic spectrum and phenotypic variations of this disease. To investigate the similarities in clinical features and metabolic pathways between the two genes and other non-isolated AN genes, as well as their potential pathogenesis, we analyzed the shared cellular components, biological processes, and molecular functions of non-isolated AN genes. Additionally, we reviewed the clinical phenotypes of typical syndromes associated with these genes. This study provides a theoretical foundation for further research into the mechanisms of mitochondrial energy metabolism and potential therapeutic approaches for non-isolated AN.

## Methods

### Participants and clinical evaluations

This study was approved by the Committee of Medical Ethics of Chinese People’s Liberation Army General Hospital(No. S2020-228-01) and conducted according to the Declaration of Helsinki. We obtained written informed consents from all the participants in this study. Seven independent Han patients were recruited, including two with *FDXR* gene mutations (one male, Patient 1, and one female, Patient 2) and five with *TWNK* gene mutations (three males, Patient 3–5, and two females, Patient 6 and Patient 7). All participants underwent ABR and distortion product otoacoustic emissions (DPOAE) as the diagnostic examinations. Additional routine examinations included pure tone audiometry (PTA), acoustic immittance, speech discrimination scores (SDS), electrocochleography (ECochG), cochlear microphonic (CM), 40 Hz auditory evoked response potential (40 Hz AERP) and inner auditory canal magnetic resonance imaging (MRI). For patients with other systemic clinical phenotypes, we conducted corresponding examinations like visual field testing, fundoscopy, optical coherence tomography and so on. We conducted long-term follow-ups with the patients through face-to-face consultations at the hospital and telephone interviews. The follow-ups utilized standardized questionnaires, assessing auditory and speech abilities using categories of auditory performance (CAP) and speech intelligibility rating (SIR) classifications, and documented any new symptoms that appeared during the course of the disease [20, 21].

### Genetic sequencing and variant assessment

We collected the peripheral blood samples from seven patients and their parents using EDTA anticoagulant tubes and extracted the genomic DNA using the Blood DNA kit (TIANGEN BIOTECH, Beijing, China). Trio-based whole exome sequencing (WES) and proband-only WES were then conducted for genetic sequencing, as described in detail previously [7, 9, 22]. Subsequently, Sanger sequencing was utilized to validate the candidate variants obtained from high-throughput sequencing.

### Bioinformatics analysis and variant pathogenicity assessment

High-frequency variants with an allele frequency greater than or equal to 0.01 in the normal population were eliminated, according to several large-scale population-based databases, including 1000 Genome Project, Exome Sequencing Project 6500 (ESP6500) and gnomAD. Bioinformatics analyses were carried out using the following tools, including Rare Exome Variant Ensemble Learner (REVEL), Sorting Intolerant From Tolerant (SIFT), Polymorphism Phenotyping version 2 (PolyPhen-2), Likelihood Ratio Test (LRT), MutationTaster, Phylogenetic P-Value (Phylop), Genomic Evolutionary Rate Profiling_Rejected Substitution (GERP++_RS) to predict the function of potential pathological variants (last accessed on April 8, 2024).

We subsequently examined the conservation of the amino acid variants discovered in this study for ferredoxin reductase and Twinkle protein across different species using Uniprot and MutationTaster. We then annotated all reported *FDXR* and *TWNK* variants using IBS1.0.3 [23], and labeled the spatial position of amino acid variants on the protein structure using PyMOL. Finally, each variant was classified based on the American College of Medical Genetics and Genomics and the Association for Molecular Pathology (ACMG/AMP) criteria [24, 25] as pathogenic (P), likely pathogenic (LP), variants with uncertain significance (VUS), likely benign (LB), and benign (B).

### Enrichment analysis of genes in non-isolated AN

Two physicians with extensive experience in genetics and otology independently conducted a search and review of articles related to non-isolated AN genes using PubMed, Web of Science, and Embase databases. The search terms included “non-isolated auditory neuropathy”, “syndromic auditory neuropathy”, “syndrome”, “auditory neuropathy”, “gene”, “mutation” and “genetic factors”. Employing a combination search strategy, we aimed to retrieve all relevant studies without restrictions on language or publication status, up to June 2024. The literature included needed to have at least an English title and/or abstract and was subsequently cross-checked. After importing all references into EndNote and removing duplicates, two reviewers independently screened the titles and abstracts. Additionally, the references of all retrieved articles were manually reviewed. The inclusion criteria required patients to be confirmed as having auditory neuropathy through audiological examination and to carry genes identified as pathogenic for the syndromic phenotype. The specific genes included in the study were determined based on the consensus of the two physicians.

Subsequently, we summarized the phenotypic characteristics of all non-isolated AN genes and conducted enrichment analysis of the candidate genes using the database established by the Gene Ontology Consortium, examining the genes from three perspectives: biological process (BP), cellular component (CC), and molecular function (MF). The analysis was performed using the R 4.2.3 software and the clusterProfiler package. To visualize the results, we employed an online data analysis and visualization platform (https://www.bioinformatics.com.cn, last accessed June 4, 2024) to generate Sankey and bubble plots, incorporating the top 10 enriched pathways from each perspective.

## Results

### General characteristics

**Fig. 1:**
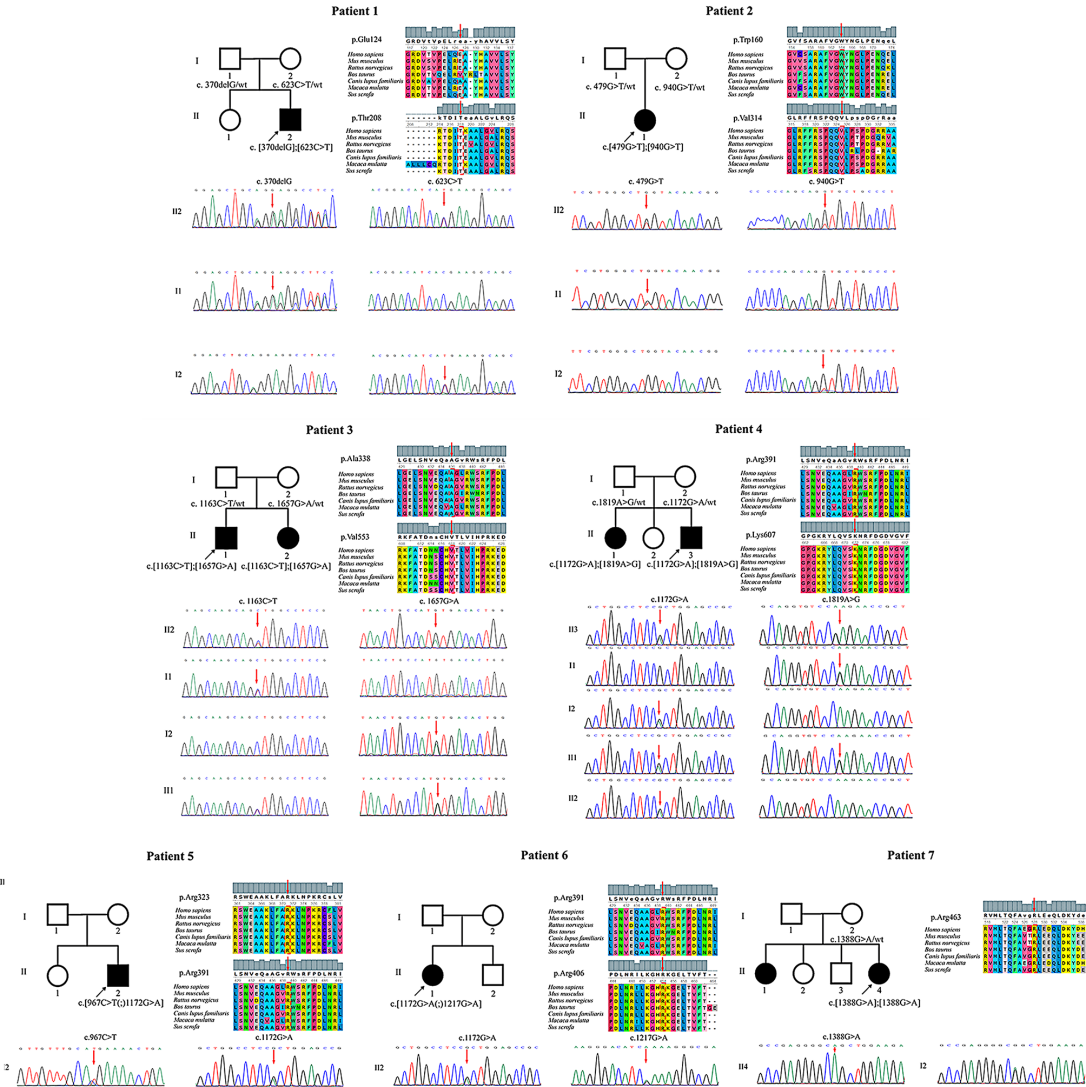
Pedigree, Sanger sequencing and interspecies sequence comparison of the seven families included in this study. *FDXR* variants are reported according to GenBank: NM_024417.5. *TWNK* variants are reported according to GenBank: NM_021830.5. Black arrows indicate the probands, red boxes represent the positions of the mutated amino acids and red arrows point to mutant bases

A total of 7 Han patients (4 males and 3 females) with non-isolated AN from 7 families were included in this study (Fig. 1). Hearing loss was the initial symptom in all but one patient (Patient 6), who had congenital urinary system malformations. The age of onset for hearing loss ranged from 2 to 25 years, with a mean age of onset of approximately 11 years (11.29 ± 7.23). Four of seven patients (57.1%) had other risk factors for hearing loss. Patient 1 had a normal birth history but experienced head trauma and noise exposure during youth. Patient 2 was born prematurely at eight months of pregnancy with a low birth weight. Two days after birth, she developed symptoms of hematemesis and hematochezia, necessitating NICU hospitalization. Patient 4 had a medical history of epilepsy, premature rupture of amniotic fluid, and required oxygen therapy. Patient 5 had a medical history of hypoxic encephalopathy and neonatal upper respiratory tract infection.

### Hearing evaluation and imaging examination

#### Subjective tests

**Fig. 2:**
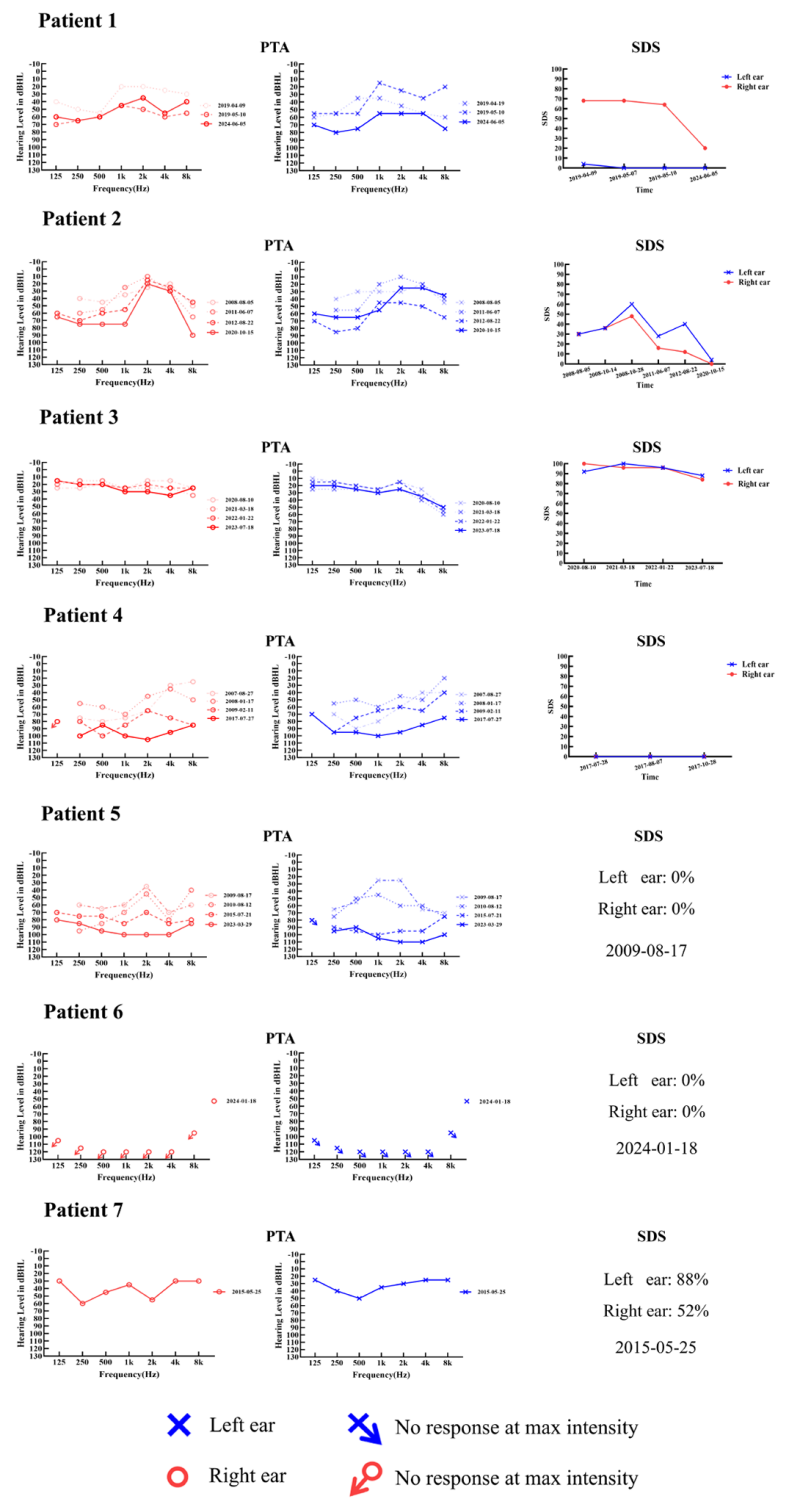
The characteristics of the subjective auditory tests of the patients. PTA: pure-tone audiometry, SDS: speech discrimination score

All patients exhibited varying degrees of hearing threshold decline, ranging from mild loss to complete deafness. The types of audiometric curves observed included ascending, flat, descending, and irregular patterns. Their speech recognition abilities were generally poorer than their hearing thresholds level. (Fig. 2). Both patients with *FDXR* mutations exhibited bilateral AN, with pure tone audiometry indicating moderate to moderately severe hearing loss. In contrast, patients with *TWNK* mutations showed a wide range of hearing levels, from mild impairment to complete deafness. Patient 3 had nearly normal hearing, with only difficulty hearing in noisy environments, while Patient 6 was diagnosed with severe hearing loss in early years; all other patients demonstrated post-lingual, symmetrical, and bilateral sensorineural hearing loss.

#### Objective tests

**Fig. 3:**
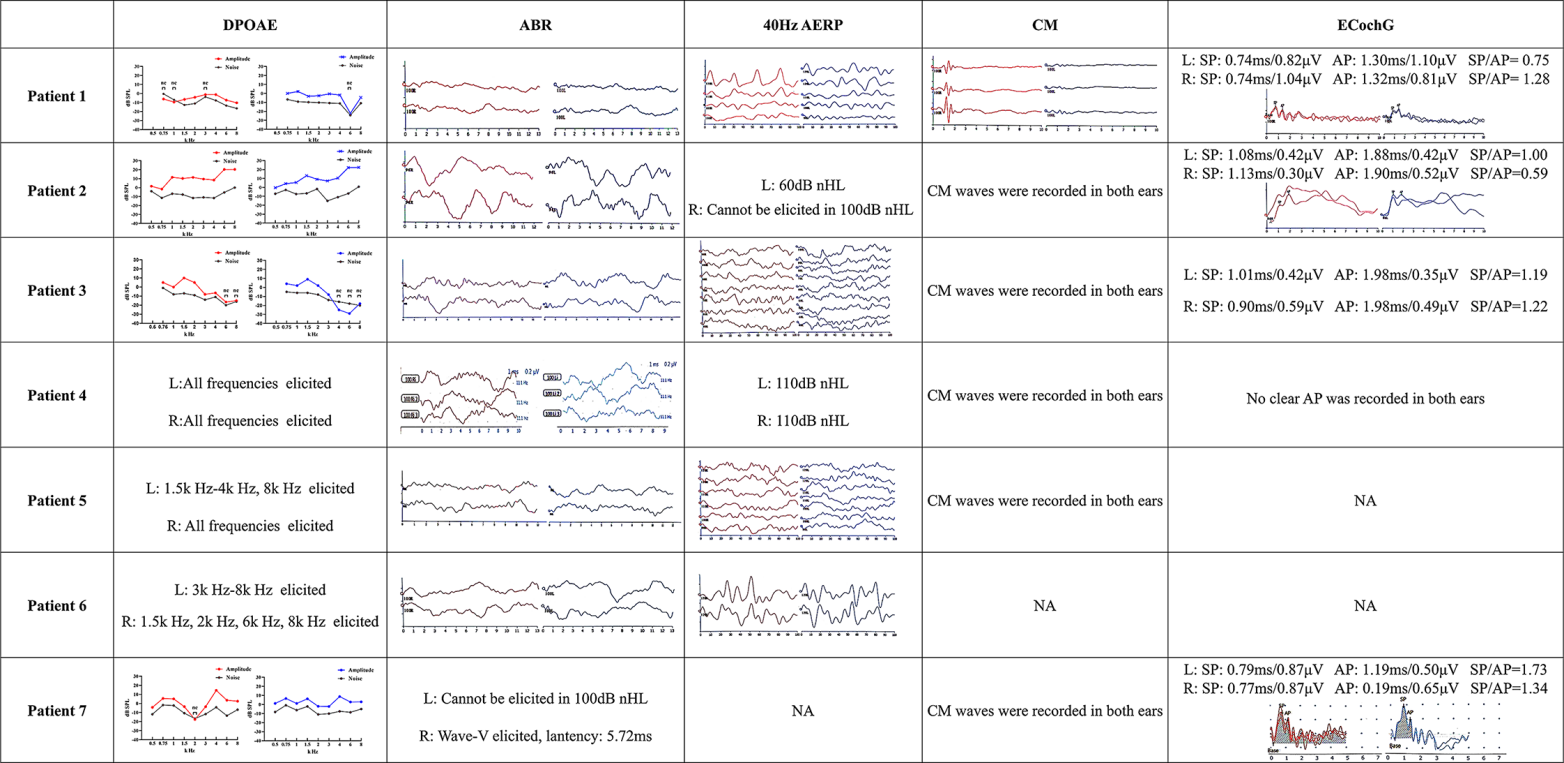
The characteristics of the objective auditory tests of the patients. DPOAE: distortion product otoacoustic emissions, ABR: auditory brainstem response, EcochG: Electrocochleography, CM: cochlear microphonic, 40 Hz AERP: 40 Hz Auditory Evoked Response Potential. SP: summating potential, AP: action potential. NA: not available

All patients underwent acoustic immittance tests and conductive problems were excluded. For the diagnostic audiological examination of AN, auditory brainstem response (ABR) waveforms could not be elicited, but distortion product otoacoustic emissions (DPOAE) could be elicited in the early stages of the disease. Among all patients, CM waves were elicited in 86% of cases. Additionally, 71% of patients underwent ECochG testing. Of these, one patient showed no discernible action potential (AP) in both ears, while the remaining patients exhibited a reduced AP amplitude, resulting in an elevated summating potential (SP)/AP ratio with a mean of 1.00 ± 0.47. Across the entire patient group, the average SP amplitude was 0.67 ± 0.25µV, the average SP latency was 0.90 ± 0.13 ms, the average AP amplitude was 0.61 ± 0.23µV, and the AP latency averaged 1.47 ± 0.58 ms. Furthermore, 86% of the patients exhibited elevated 40 Hz AERP thresholds (Fig. 3).

#### MRI

**Fig. 4:**
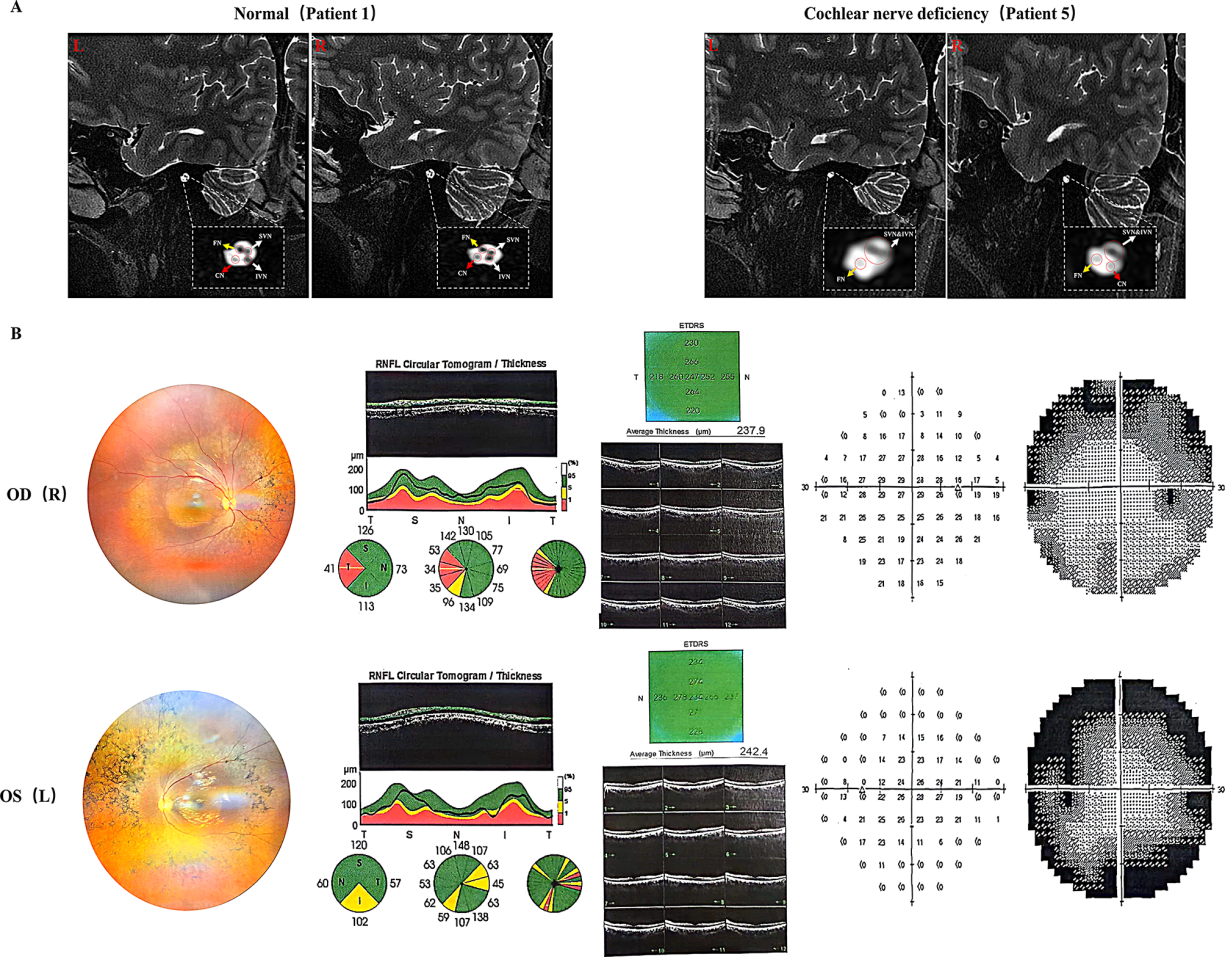
Other examination results of some patients. A: MRI images of normal auditory nerves and cochlear nerve deficiency in patients. The yellow arrow points to the facial nerve, the white arrows point to the superior and inferior vestibular nerves, and the red arrow points to the auditory nerve. In Patient 5, the left auditory nerve is unclear. B: Ophthalmologic examination results of Patient 1

Among the 7 patients, 6 underwent MRI to observe the condition of their auditory nerves. Of these, 33.3% (Patient 4 and 5) were found to have abnormalities in their auditory nerves, and both carried mutations in the *TWNK* gene (Fig. 4A).

### Concomitant symptoms

Of all patients, 5 patients (71.4%) presented with other systemic symptoms and the clinical phenotypes exhibited significant heterogeneity. Ataxia, the most common symptom, was observed in 57.1% patients (Patient 1, 4, 5, 6). Abnormalities of the musculoskeletal system were also common symptoms, with 42.9% patients (Patient 4, 5, 6) exhibiting high arches, and 28.6% patients (Patient 4 and 6) exhibited scoliosis. Patient 1, besides being diagnosed with auditory neuropathy, was simultaneously found to have vision problems. Examination revealed a corrected visual acuity of 0.3 in both eyes, pale optic discs, bone spicule-like changes in the retinas, centripetal visual field constriction, and thinning of the ganglion cell layer in the macular area on OCT. The patient was diagnosed with optic atrophy and retinitis pigmentosa (Fig. 4B). The clinical characteristics of all patients were summarized in Table 1.

**Table 1:** Overview of patients in this study on genotype and phenotype

Patient ID	*FDXR*(NM_024417.5; NP_077728.3)		*TWNK*(NM_021830.5; NP_068602.2)				
	Patient 1	Patient 2	Patient 3	Patient 4	Patient 5	Patient 6	Patient 7
Nucleotide change	c.370delG	c.479G > T	c.1657G > A	c.1819A > G	c.967C > T	c.1217G > A	c.1388G > A
	c.623C > T	c.940G > T	c.1163C > T	c.1172G > A	c.1172G > A	c.1172G > A	c.1388G > A
Amino acid change	p.Glu124Argfs*8	p.Trp160Leu	p.Val553Met	p.Lys607Glu	p.Arg323*	p.Arg406Gln	p.Arg463Gln
	p.Thr208Met	p.Val314Leu	p.Ala388Val	p.Arg391His	p.Arg391His	p.Arg391His	p.Arg463Gln
Gender	M	F	M	M	M	F	F
Age	24	31	31	23	21	20	26
Onset of hearing loss	16	10	25	6	6	2	14
Impaired vision	Onset at 16	-	-	-	-	-	-
Pes cvus	-	-	-	+	Onset at age 8	+	NA
Scoliosis	-	-	-	Onset at age 12	+	+	NA
Ataxia	Onset at age 24	-	-	Onset at age 12	Onset at age 5	+	NA
Urinary malformation	-	-	-	-	+	Congenital	NA
Abnormal cochlear nerve	-	-	+	+	-	-	NA
Muscle weakness	Onset at age 24	Onset at age 31	-	NA	+	NA	NA

M: male, F: female, +: The symptom has already appeared, -: The symptom has not been observed yet, NA: not available

### Follow-ups

During our follow-ups, excluding Patient 7 who was lost to follow-up, the remaining six patients had an average follow-up period of 9.33 ± 4.37 years, with the shortest follow-up being 3 years and the longest being 12 years. Patient 6 had almost no residual hearing at her first visit, making it difficult to assess any further hearing decline over time. The remaining 5 patients experienced varying degrees of hearing loss over time, with an average hearing threshold decline rate of 2.35 ± 0.96 dB HL per year across all frequencies. This decline averaged 2.62 ± 1.28 dB HL per year in patients with *FDXR* mutations and 1.84 ± 0.64 dB HL per year in those with *TWNK* mutations. Compared to the initial consultation, four patients exhibited new symptoms. Patient 1 exhibited symptoms of unstable gait and frequent falls while running at the age of 24. He had a CAP level of 7 and an SIR level of 5. Patient 2 exhibited symptoms of decreased chewing ability and facial pain at the age of 31. Regarding her hearing, she had a CAP level of 3 and an SIR level of 5. Patient 5 underwent bilateral osteotomy and internal fixation for foot and ankle deformity, along with urethral stricture dilation at the age of 18. Due to poor hearing since childhood, Patient 6 underwent a cochlear implantation in the right ear at the age of 7. She experienced an unexplained sudden increase in lower limb weakness at 16, leading to an inability to walk upright. After using the cochlear implant for 12 years, she had it removed due to its limited benefit to her hearing, with subsequent follow-up indicating a CAP level of 2 and an SIR level of 1.

### High-throughput sequencing and Sanger sequencing

Through high-throughput sequencing and Sanger sequencing, we identified a total of four *FDXR* variants in two patients, three of which were novel. Among these four variants, three were missense variants (p.Thr208Met, p.Trp160Leu and p.Val314Leu) located in the flavin adenine dinucleotide/nicotinamide adenine dinucleotide (phosphate) (FAD/NAD[P])-binding domain, while one was a frameshift mutation (p.Glu124Argfs*8) located in the NAD (P)-binding domain.

**Fig. 5:**
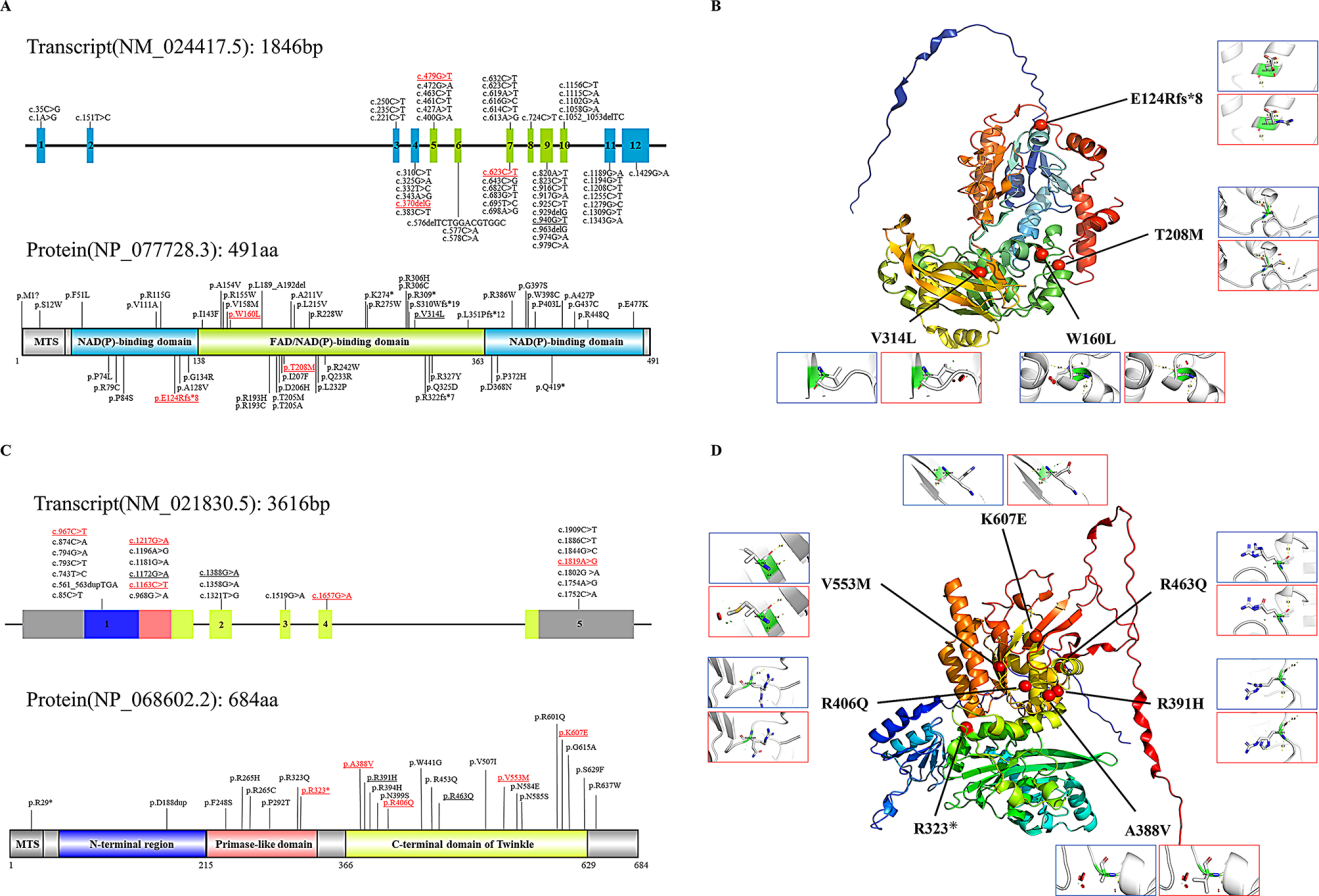
Mutations and Protein Product of *FDXR* and *TWNK* gene in this study and previous studies. **A**. Structure of the *FDXR* gene and its predicted protein product. **B**. 3D structure of the FDXR protein with annotated mutated amino acid positions from this study. **C**. Structure of the *TWNK* gene and its predicted protein product. **D**. 3D structure of the Twinkle protein with annotated mutated amino acid positions from this study. Exons are represented by numbered boxes. The mitochondrial targeted sequence (MTS) is shown in gray, the NAD(P)-binding domain in lemon, the FAD/NAD(P)-binding domain in sky blue, the N-terminal region in dark blue, the Primase-like domain in pink, and the C-terminal domain of Twinkle in yellow-green. For base and amino acid changes, black represents previously reported mutations, underlined indicates mutations found in patients in this study, and red denotes novel mutations. The 3D structure of the protein transitions from blue at the N-terminal region to red at the C-terminal region. Mutated amino acid positions are marked with red spheres, while the spatial structures of the original and mutated amino acids are indicated by blue and red boxes, respectively

Five patients with *TWNK* gene mutations were included in this study, comprising four patients with compound heterozygous variants and one patient with a homozygous variant. A total of seven *TWNK* missense variants were identified, five of which were novel. Six variants (p.Ala388Val, p.Arg391His, p.Arg406Gln, p.Arg463Gln, p.Val553Met and p.Lys607Glu) were in the C-terminal domain of Twinkle, and p.Arg323*, resulting in premature termination codon was identified within the primase-like domain (Fig. 5).

### Variant detection and analysis

All variants were in highly conserved residues and had a genotype frequency of less than 0.001 in the 1KGP, ESP6500, and gnomAD databases. For the four variants of *FDXR*, the p.Glu124Argfs*8 frameshift mutation induced premature termination and triggered nonsense-mediated mRNA decay and was classified as LP. The p.Val314Leu variant, appeared in the trans position of multiple disease-causing mutations in previous patients and was classified as pathogenic. The p.Thr208Met and p.Trp160Leu variants, which were in trans position relative to the reported mutations, were predicted to be deleterious by all bioinformatic tools, and classified as LP. The *TWNK* c.967 C > T variant resulted in the production of a premature termination codon, leading to p.Arg323*, and was classified as P. The remaining six variants (p.Ala388Val, p.Arg391His, p.Arg406Gln, p.Arg463Gln, p.Val553Met and p.Lys607Glu) were missense mutations and were considered LP. Genotype frequency, software prediction results, and ACMG classification for each variant were provided in Supplementary Tables 1–2.

### Non-isolated AN genes function analysis

**Fig. 6:**
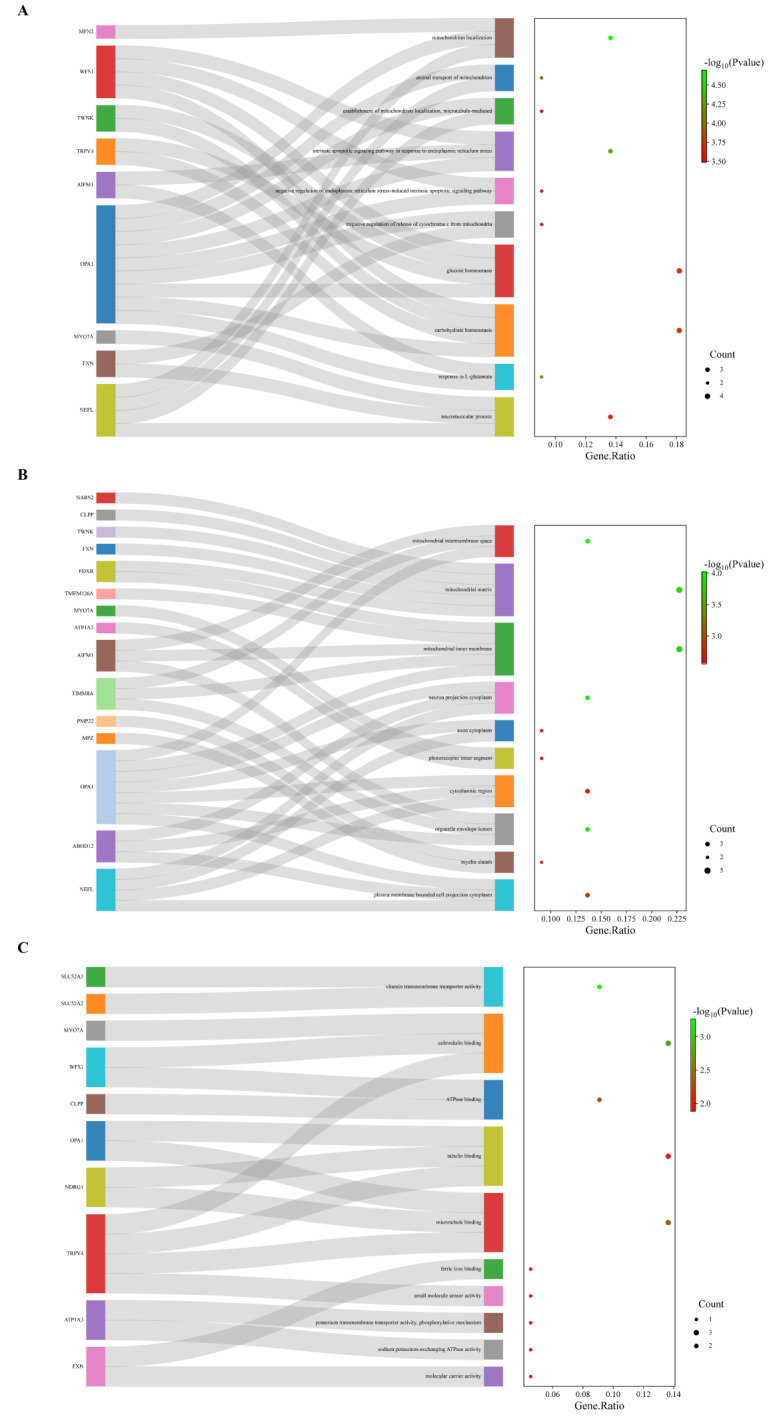
Non-isolated AN genes function analysis: **A**. The top 10 enriched pathways of biological process. **B**. The top 10 enriched pathways of cellular component. **C**. The top 10 enriched pathways of molecular function

Through a comprehensive review of published literatures, we identified 22 genes that were definitively associated with the pathogenesis of non-isolated AN. Among all syndromes, the commonly affected aspects are the central nervous system, peripheral nervous system, musculoskeletal system, visual system, and development. The clinical phenotypes linked to these genes were detailed in Table 2 and Supplementary Table 3. We conducted a functional enrichment analysis on these genes, revealing that the implicated biological processes predominantly involve mitochondrial localization and transport, stress response, regulation of apoptosis, metabolic processes, homeostasis maintenance, as well as neurological and muscular functions. The cellular components were chiefly enriched in mitochondrial and neuronal components, while the molecular functions were significantly associated with transport activity, protein binding, molecular carrier activity, ion homeostasis, and transmembrane transport (Fig. 6).

**Table 2:** Summary of clinical symptoms associated with non-isolated AN-related genes

Disease	Gene	AN	Central nervous system	Peripheral nervous system	Musculoskeletal	Eyes	Development	Others
ANOA	*FDXR*	+	+	+	+	+	+	
Autosomal-dominant optic atrophy (DOA)	*OPA1*	+	+	+	+	+	+	Thyroid dysfunction
Brown-Vialetto-Van Laere syndrome 1	*SLC52A2*	+	+	+	+	+	+	
	*SLC52A3*	+	+	+	+	+	+	
CAPOS syndrome	*ATP1A3*	+	+	+	+	+	+	Cardiogenic syncope, Autism spectrum disorder
Charcot-Marie-Tooth (CMT) syndrome	*MPZ*	+	+	+	+	+	+	Gastrointestinal symptoms, erectile dysfunction, uroclepsia
	*PMP22*	+	+	+	+	-	+	
	*NDRG1*	+	+	+	+	+	+	
	*NEFL*	+	+	+	+	+	+	
	*MFN2*	+	+	+	+	+	+	
	*TRPV4*	+	+	+	+	-	+	Vocal cords paralysis
	*ABHD12*	+	+	+	+	+	+	
Cowchock syndrome	*AIFM1*	+	+	+	+	+	+	
Friedreich ataxia	*FXN*	+	+	+	+	+	+	Cardiomyopathy, left ventricular hypertrophy, diabetes mellitus
Leber hereditary optic neuropathy (LHON)	*TMEM126A*	+	-	+	+	+	-	Renal tubular acidosis, arrhythmia, cardiomyopathy
Leigh syndrome	*NARS2*	+	+	+	+	-	+	
Mohr-Tranebjaerg syndrome	*TIMM8A*	+	+	+	+	+	+	
Perrault syndrome	*TWNK*	+	+	+	+	+	+	Thyroid dysfunction, ovarian dysfunction, primary amenorrhea, gonadal dysgenesis
	*CLPP*	+	+	+	+	-	+	
Usher syndrome	*MYO7A*	+	-	-	+	+	+	Vestibular dysfunction
White-Sutton syndrome	*POGZ*	+	+	-	+	+	+	Autism spectrum disorder, atrial septal defect, hypospadias
Wolfram syndrome	*WFS1*	+	+	-	-	+	-	Diabetes mellitus, mental illness, abnormal urinary system, autoimmune diseases

+: The symptom has already observed, -: The symptom has not been observed yet

## Discussion

AN can occur independently or be accompanied by symptoms affecting other systems, such as central or peripheral neuropathy, with the latter accounting for about one-third of AN cases [5]. The interaction mechanisms between hearing impairments and systemic diseases are complex, contributing to significant clinical heterogeneity. Given the dual influence of genetic and environmental factors on AN, it is imperative to identify its genetic underpinnings. The advancement of next-generation sequencing technologies has greatly improved diagnostic capabilities, aiding in the early identification of patients carrying pathogenic variants [26]. In this study, we focused on two non-isolated AN-related genes, *FDXR* and *TWNK*, and analyzed patients’ clinical phenotypes and mutation sites to explore the pathogenesis of non-isolated AN and its mechanism.

### Identification of pathogenic variants and their association with phenotypes

For *FDXR* gene, previously reported mutations are primarily located in the FAD/NAD (P)-binding domain, indicating that this domain plays a crucial role in the normal function of the *FDXR* protein. In this study, two newly identified missense mutations (p.Thr208Met and p.Trp160Leu) and one previously reported mutation (p.Val314Leu) are also located in this domain. The p.Val314Leu (rs763331014) mutation, which is the most frequently reported mutation in the *FDXR* gene to date with five documented cases [27–29], has well-established pathogenicity, making it a hotspot mutation in the *FDXR* gene. However, in all five previously reported cases, the patients presented with normal hearing at their initial diagnosis, marking this study as the first report of AN associated with this specific mutation. The p.Glu124Argfs*8 mutation results in the deletion of a nucleotide, which changes the position of the stop codon, causing premature termination of translation and a reduction in protein molecular weight. This mutation occurs in the first NAD (P)-binding domain of the *FDXR* protein, leading to the loss of function in the FAD/NAD (P)-binding domain and the second NAD (P)-binding domain, resulting in the loss of key functional domains of the *FDXR* protein, which may severely impact the protein’s function.

Unlike previously reported phenotypes of patients with *FDXR* gene mutations, our patients presented with AN as the initial symptom. Both patients experienced a decline in speech recognition ability in their teenage years. During subsequent follow-ups, we observed peripheral nerve involvement in both patients, primarily manifesting as muscle weakness. Among the two patients, Patient 1 was diagnosed with optic atrophy through ophthalmologic examination, while Patient 2 did not exhibit any vision problems over a 16-year follow-up period, which contrasted with the previously reported 93.2% (55/59) incidence rate of optic atrophy in auditory neuropathy and optic atrophy patients [28]. Consistent with previous cases of auditory neuropathy and optic atrophy(OMIM: 617717), our patients also exhibited symptoms affecting at least two systems. However, compared to the significant heterogeneity in the clinical manifestations of AN caused by *FDXR* mutations, our patients exhibited fewer clinical symptoms [30–33]. It remains to be determined whether this is due to milder clinical presentations or the absence of other systemic symptoms that may not have emerged yet, which requires further follow-ups and additional diagnostic tests.

In this study, a total of seven *TWNK* variants were identified, five of which were newly reported in connection with Perrault syndrome caused by *TWNK* gene mutations. Four mutations were found on exon 1 of *TWNK* gene, while t he remaining three were located at exon 2, 4, and 5. Six of the encoded amino acids were situated within the C-terminal domain of Twinkle, and p.Arg323* was located within the primase-like domain. The C-terminal domain influences helicase activity, DNA-binding ability, as well as subunit interaction or stability. Mutations in this region can lead to reduced enzyme activity and impede mitochondrial DNA replication [34, 35]. Mutations in the primase-like domain can significantly reduce ATPase activity and cause damage to single-stranded DNA Primase-like domain [36].

Different combinations of alleles resulting from *TWNK* compound homozygous or heterozygous mutations, along with the interaction of other modified genes and environmental factors, can lead to various symptoms in Perrault syndrome with different severity. Patient 3 carried *TWNK* c.[1657G > A]; [1163 C > T] mutations and exhibited only mild high-frequency hearing loss with nearly normal speech recognition rate. Typically, this kind of hearing level would be associated with normal speech recognition. Patient 3’s speech discrimination score was unusually below 90%, which aligns with an audiometric profile often observed in AN. Also, this patient did not exhibit any systemic symptoms, possibly due to the minimal impact of mutations on protein function or compensatory mechanisms, or he was in an early stage of AN. Patient 5 carried the p.Arg323* mutation, which resulted in a stop-gain variation within the Primase-like domain. This patient exhibited hearing loss at the age of 6, along with clinical manifestations such as ataxia, pes cavus, and urinary tract stenosis. These findings suggest that the mutation has a significant functional impact on the Twinkle protein. Three patients carried *TWNK* p.Arg391His mutation and they experienced an early onset of severe to profound hearing loss at the age of 2–6 years old, accompanied by systemic manifestations, suggesting that the presence of this mutation may serve as a predictor for poor outcome. This mutation had previously been reported in two Chinese families and one Japanese family, with patients exhibiting an age of onset ranging from 3 to 13 years, indicating a hotspot mutation. In Patient 7, a homozygous p.Arg463Gln mutation in the *TWNK* gene was identified. She presented with symmetric moderate hearing loss in both ears. While her speech discrimination score in the left ear was relatively normal, her right ear showed a significant decline, with the score dropping to nearly 50%, indicating a disproportionate impact on speech comprehension in that ear. This mutation had also been identified in a Chinese family, where affected individuals exhibited homozygous mutations with disease onset at ages 20 and 22, respectively. Experiment in vitro showed *TWNK* p.Arg463Gln impaired mitochondrial mtDNA replication and respiratory potential, but ROS level was not affected [37].

Previous studies showed that patients with *TWNK* gene mutations primarily exhibit hearing loss and high arched feet, while female patients often experience ovarian dysgenesis and other gonadal dysfunctions [38–40]. Additionally, the condition may be accompanied by central and peripheral nervous system symptoms, such as ataxia and nystagmus [12, 41, 42]. Typical symptoms were observed in our patients as well, but female reproductive system abnormalities have not been discovered so far, which may be due to the small number of patients or varying degrees of pathogenicity. Patient 2, 4 and 5 had high-risk factors in neonatal period, which may be related to the severe phenotype of the patients, indicating that the existence of high-risk factors may aggravate the progression and severity of the disease.

Objective electrophysiological audiometry offers valuable insights for diagnosing AN. In this study, five patients (71.4%) underwent ECochG; among them, AP was unrecordable in one patient, while both SP and AP were recorded in the remaining four. No significant changes were observed in SP latency, AP latency, or SP amplitude across these patients. However, a marked reduction in AP amplitude was noted, leading to an elevated SP/AP ratio. Previous studies [43, 44] on ECochG in AN patients have reported that the SP/AP ratio is often abnormal, frequently exceeding 1, which aligns with the findings in our study. The SP reflects the receptor potentials of hair cells, while the AP response indicates postsynaptic status. Studies on ECochG in AN patients have shown that those with postsynaptic AN typically exhibit a normal SP but an absent or reduced AP amplitude [45–47]. Based on the phenotypes observed in our patient group, the primary affecting site in patients with *FDXR* and *TWNK* mutations was post synapse.

It is noteworthy that two of six patients (33.3%) underwent MRI examination in this study had cochlear nerve deficiency, indicating that a significant proportion of patients with non-isolated AN may have detectable structural anomalies in the auditory nerve, particularly among those with *TWNK* gene mutations. This underscores the importance of genetic screening and imaging studies in the comprehensive diagnostic evaluation of these patients.

### *FDXR* and *TWNK* genes play crucial roles in mitochondrial function

Both the cochlear stria vascularis and neurons in the central auditory pathways are highly sensitive to energy deficiencies, making normal mitochondrial function essential for normal hearing. Abnormalities in mitochondrial DNA or any part of the oxidative respiratory chain can impair mitochondrial function. Previous studies have shown that many non-isolated AN-related genes, such as *AIFM1*, *TIMM8A*, *ATP1A3* and *OPA1*, are closely linked to mitochondrial function [8–11, 48]. Mitochondrial diseases are a group of genetic disorders characterized by defects in oxidative phosphorylation. Mitochondrial diseases, one of the most common hereditary metabolic and neurological disorders, exhibit significant clinical heterogeneity. Approximately 1500 proteins are associated with mitochondrial function, but only about 100 of these are closely related to the oxidative phosphorylation process [49, 50]. In this study, we focus on two crucial genes, *FDXR* and *TWNK*, to further understand their roles in mitochondrial function and their potential link to non-isolated AN.

The *FDXR* gene encodes ferredoxin reductase, which acts as the primary electron transfer protein in all mitochondrial P450 systems, playing a crucial role in electron transfer, iron-sulfur cluster synthesis, and the functioning of cytochrome P450. It is regulated by the p53 family, with the p53 protein binding to response elements on the *FDXR* promoter to regulate its expression, thereby influencing cell cycle arrest and apoptosis [51, 52]. Mutations in the *FDXR* gene impair the biosynthesis of Fe-S clusters, which compromises the function of various iron-sulfur proteins and disrupts mitochondrial activity, leading to energy metabolism disorders, the accumulation of reactive oxygen species (ROS), and subsequent oxidative stress-induced cellular damage [53]. Neurons have exceptionally high demands for energy and metabolic stability, and any defects in mitochondrial function can lead to their degeneration and death, resulting in clinical symptoms such as sensorimotor, optic, and auditory neuropathy [54]. *FDXR* deficiency caused by gene mutations is an autosomal recessive mitochondrial disorder and is considered one of the 50 most prevalent mitochondrial diseases [55].

The *TWNK* gene encodes the Twinkle protein, comprising a primase-related domain, a helicase domain, and an intermediate linker region. Mutations in the primase-related domain can significantly reduce ATPase activity and impair single-stranded DNA binding [36]. The helicase domain mutations affect helicase activity, DNA binding capability, and interactions or stability with subunits, leading to decreased enzyme activity and stalling mitochondrial DNA replication [34, 35]. The linker region is crucial for oligomerization and helicase activity [56, 57]. The Twinkle protein plays a critical role in mtDNA replication by unwinding the mtDNA replication fork, ensuring smooth DNA replication. The function of the Twinkle protein is essential for the lifelong maintenance of human mitochondria. Mutations can lead to mitochondrial DNA depletion or deletions, causing various mitochondrial diseases.

These two genes play crucial roles in maintaining mitochondrial function, participating in core biological processes within mitochondria to ensure their proper operation and maintain stability in cellular energy metabolism. Mutations in these genes can disrupt energy metabolism and induce oxidative stress, ultimately leading to cellular damage and neurodegenerative diseases. These genetic defects not only affect energy-demanding neurons but also manifest clinically as various mitochondrial-related disorders such as sensorimotor, optic, and auditory neuropathies.

### The role of mitochondrial function in the pathogenesis of non-isolated AN

Gene enrichment analysis of non-isolated AN revealed significant commonality in various biological processes and pathways, primarily centered on mitochondrial function and neural activity. These genes were prominently associated with mitochondrial components such as intermembrane spaces, stroma, and inner mitochondrial membranes, as well as neuronal structures including axons, dendrites, and photoreceptor inner segments. They played crucial roles in mitochondrial metabolism, oxidative phosphorylation, neurotransmission, synaptic function, and visual signaling. Biologically, non-isolated AN-related genes showed notable enrichment in processes related to mitochondrial localization and transport, stress response, apoptosis regulation, energy metabolism, nerve signal transmission, and neuromuscular function. At the molecular level, these genes participated in diverse activities such as vitamin transmembrane transport, protein binding with calmodulin, ATPase, microtubule, and tubulin, as well as molecular carrier functions involving sodium-potassium exchange ATPase activity and potassium ion transmembrane transport. Additionally, they were involved in small molecule sensor activity, enabling them to sense and respond to changes in cellular and extracellular small molecules. These findings underscored the critical role of non-isolated AN-related genes in neuronal metabolism, stress response, energy homeostasis, and signal transmission. Non-isolated AN as a potential neurodegenerative disorder.

Degenerative neurological diseases are a group of disorders characterized by the gradual dysfunction or death of central and peripheral nervous system cells, which manifest as a progressive loss of neuronal structure or function over time, ultimately leading to cell death. Their pathogenesis is closely related to factors such as inadequate energy supply, excessive reactive oxygen species, disrupted calcium ion homeostasis, impaired mitochondrial autophagy, and apoptosis. The role of mitochondria in neurodegenerative diseases is increasingly recognized [58]. Neurons heavily rely on mitochondrial energy production to maintain normal function and survival; thus, mitochondrial dysfunction can lead to energy insufficiency and subsequently trigger neurodegeneration [59].

In typical neurodegenerative diseases such as Alzheimer’s disease, significant deficits in central auditory processing and auditory scene analysis have been observed [60, 61]. The main pathogenic views of non-isolated AN are denervation and neuronal desynchronization, involving mitochondrial energy metabolism disorders, synaptic transmission impairment, neuronal demyelination, and apoptosis. These views suggest that non-isolated AN may essentially be a neurodegenerative disease, with changes in hearing potentially serving as specific markers for detecting neurodegenerative diseases [62].

### Limitations and future directions

Firstly, because of the rarity of this disease, the sample size in our study was relatively small, which may affect the generalizability of our findings. Furthermore, we were unable to explain some phenotypic differences from previous studies, which may be due to individual case specificity. Lastly, for non-isolated AN-related genes, we used strict audiological examination results as inclusion criteria, which excluded some suspected cases reported by doctors in other departments.

Until now, isolated AN and non-isolated AN are sometimes indistinguishable based on clinical presentation alone, making it difficult to determine whether patients presenting solely with AN or other systemic symptoms that have not yet manifested. Conducting numerous tests based solely on clinical observation can also increase the financial burden on patients. Gene detection is undoubtedly an effective way for the early diagnosis of this disease. Future research will focus on elucidating the pathogenic mechanisms of non-isolated AN, leveraging large-scale genomic studies and functional experiments to validate new mutations’ pathogenicity and identify potential therapeutic targets. In addition, long-term clinical follow-ups of patients with specific mutations are essential to expand our understanding of the phenotype and monitor long-term hearing changes in patients with non-isolated AN. The effectiveness of cochlear implantation as an intervention for this disease also requires further investigation.

Regarding potential therapeutic approaches, gene therapy has made significant progress in recent years [63, 64]. However, for non-isolated AN, research is still at the cellular and animal experimental stages and requires further exploration. Additionally, given the critical role of mitochondrial dysfunction in the pathogenesis of non-isolated AN, therapeutic strategies that enhance mitochondrial function may offer a promising path for treatment. Interventions that support mitochondrial health, such as reducing oxidative stress, and improving cellular energy metabolism, have shown some efficacy in preliminary studies. Specifically, certain antioxidant treatments like NADH and taurine have demonstrated beneficial effects [65, 66].

## Conclusion

This study focuses on two typical genes affecting mitochondrial function related to non-isolated AN, *FDXR* and *TWNK*, and discovers eleven variants, including eight novel pathogenic or likely pathogenic mutations. By combining these findings with the patients’ clinical data, we expand the phenotypic and genetic spectrum of these two genes. Through literature review and gene enrichment analysis, we summarized the syndromes related to non-isolated AN, which helped clarify the pathogenic mechanisms and established the genotype-phenotype correlations of non-isolated AN. Genetic testing should become an early diagnostic tool for non-isolated AN in the future.

## Electronic supplementary material

Below is the link to the electronic supplementary material.

**Supplementary Material 1: Supplementary Table 1.** *FDXR* and *TWNK* missense mutations in this study. **Supplementary Table 2.** The classification of variants in *FDXR* and *TWNK* identified in our study based on ACMG/AMP criteria. **Supplementary Table 3.** Summary of clinical symptoms associated with non-isolated AN-related genes in detail

**Supplementary Material 2:** Hearing tests of patients in this study

**Supplementary Material 3:** Non-isolated AN genes and enrichment analysis in this study

## Data Availability

The datasets generated in this study are included in the article. Due to concerns regarding patient confidentiality and participant privacy, the genotyping data are not available for public access. For inquiries or requests to access the data, please contact QW, wqjavm301@sina.com.

## Abbreviations

AN: Auditory neuropathy
OAE: Otoacoustic emission
ABR: Auditory brainstem response
IHC: Inner Hair Cell
mtDNA: Mitochondrial DNA
DPOAE: Distortion Product Otoacoustic Emissions
PTA: Pure Tone Audiometry
SDS: Speech Discrimination Scores
ECochG: Electrocochleography
SP/AP: Summating potential/Action potential
CM: Cochlear Microphonic
40Hz AERP: 40 Hz Auditory Evoked Response Potential
MRI: Magnetic Resonance Imaging
CAP: Categories Of Auditory Performance
SIR: Speech Intelligibility Rating
WES: Whole Exome Sequencing
ESP6500: Exome Sequencing Project 6500
REVEL: Rare Exome Variant Ensemble Learner
SIFT: Sorting Intolerant From Tolerant
PolyPhen-2: Polymorphism Phenotyping Version 2
LRT: Likelihood Ratio Test
Phylop: Phylogenetic P-Values
GERP++_RS: Genomic Evolutionary Rate Profiling Rejected Substitution
ACMG/AMP: American College Of Medical Genetics And Genomics And The Association For Molecular Pathology
P: Pathogenic
LP: Likely Pathogenic
VUS: Variants With Uncertain Significance
LB: Likely Benign
B: Benign
BP: Biological Process
CC: Cellular Component
MF: Molecular Function
FAD: Flavin Adenine Dinucleotide
NAD (P): Nicotinamide Adenine Dinucleotide (Phosphate)
ROS: Reactive Oxygen Species
